# Correction of pathogenic mitochondrial DNA in patient-derived disease models using mitochondrial base editors

**DOI:** 10.1371/journal.pbio.3003207

**Published:** 2025-06-24

**Authors:** Indi P. Joore, Sawsan Shehata, Irena Muffels, Jose Castro-Alpízar, Elena Jiménez-Curiel, Emilia Nagyova, Natacha Levy, Ziqin Tang, Kimberly Smit, Wilbert P. Vermeij, Richard Rodenburg, Raymond Schiffelers, Edward E.S. Nieuwenhuis, Peter M. van Hasselt, Sabine A. Fuchs, Martijn A. J. Koppens

**Affiliations:** 1 Department of Metabolic Diseases, Wilhelmina Children’s Hospital, University Medical Center Utrecht, Utrecht, The Netherlands; 2 Regenerative Medicine Center Utrecht, Utrecht, The Netherlands; 3 Princess Máxima Center for Pediatric Oncology, Utrecht, The Netherlands; 4 Oncode Institute, Utrecht, The Netherlands; 5 Department of Pediatrics, Radboud UMC, Nijmegen, The Netherlands; 6 Department of Genetics, Radboud UMC, Nijmegen, The Netherlands; 7 CDL Research, UMC Utrecht, Utrecht, The Netherlands; ETH Zurich, SWITZERLAND

## Abstract

Mutations in the mitochondrial genome can cause maternally inherited diseases, cancer, and aging-related conditions. Recent technological progress now enables the creation and correction of mutations in the mitochondrial genome, but it remains relatively unknown how patients with primary mitochondrial disease can benefit from this technology. Here, we demonstrate the potential of the double-stranded DNA deaminase toxin A-derived cytosine base editor (DdCBE) to develop disease models and therapeutic strategies for mitochondrial disease in primary human cells. Introduction of the m.15150G > A mutation in liver organoids resulted in organoid lines with varying degrees of heteroplasmy and correspondingly reduced ATP production, providing a unique model to study functional consequences of different levels of heteroplasmy of this mutation. Correction of the m.4291T > C mutation in patient-derived fibroblasts restored mitochondrial membrane potential. DdCBE generated sustainable edits with high specificity and product purity. To prepare for clinical application, we found that mRNA-mediated mitochondrial base editing resulted in increased efficiency and cellular viability compared to DNA-mediated editing. Moreover, we showed efficient delivery of the mRNA mitochondrial base editors using lipid nanoparticles, which is currently the most advanced non-viral in vivo delivery system for gene products. Our study thus demonstrates the potential of mitochondrial base editing to not only generate unique *in vitro* models to study these diseases, but also to functionally correct mitochondrial mutations in patient-derived cells for future therapeutic purposes.

## Abstract 2

Recent developments in base editing technologies enable the correction of mutations in the mitochondrial genome, but its therapeutic potential remains unclear. This proof-of-principle study shows that mitochondrial base editing can functionally create and correct mitochondrial pathogenic mutations in patient-derived cells.

## Introduction

Genetic mutations in the mitochondrial genome can cause mitochondrial dysfunction and result in aging-related conditions, certain types of cancer and debilitating and potentially fatal maternally inherited mitochondrial disease. While the development of the CRISPR/Cas technology has revolutionized possibilities for mutations encoded in the nuclear DNA, this system cannot effectively cross the mitochondrial membrane and reach mitochondrial DNA [1]. This has basically left mitochondrial genetics in a pre-genetic engineering era and mitochondrial patients unable to benefit from advancements in gene correction strategies.

Recently, the development of the double-stranded DNA deaminase toxin A (DddA)-derived cytosine base editor (DdCBE) addressed this problem and enabledthe introduction of edits in the mitochondrial genome by using a CRISPR/Cas-independent system [2]. DdCBE combines Transcription Activator-Like Effectors (TALEs) with the cytosine deaminase activity of a split version of the interbacterial toxin DddA, enabling efficient installation of TC > TT mutations with high specificity and product purity. Recent developments have improved DdCBE with phage-assisted evolution to enable more flexible context editing (AC/CC > AT/CT) [3], by nuclear exclusion to reduce nuclear off-targets [4] and by using slightly impaired DdCBE complex formation to drastically decrease mitochondrial off-target editing [5]. DdCBE enables the generation of mitochondrial disease models, for instance through creation of mitochondrial OXPHOS complex knockout libraries [6], editing human embryos [7], and generating zebrafish [8,9], and rodent [4,10] models. Mitochondrial adenosine base editors have also been developed [11,12]. While these studies have accelerated mitochondrial mechanistic research, the clinical potential of mitochondrial gene editing remains less explored. Although the introduction of mitochondrial mutations has been shown to reduce mitochondrial activity, it remains unclear whether this effect stems from the mutation or the editing process itself [12]. Studies on correction of a pathogenic mutation in primary patient-derived cells are lacking and the functional effects remain unknown. Furthermore, studies on the clinical implementation of mitochondrial base editors have so far been limited to introducing novel mitochondrial mutations through adeno-associated virus (AAV)-mediated DdCBE-delivery [13,14].

To explore the potential of clinical application of DdCBE-mediated mitochondrial DNA gene editing, we generated and functionally corrected pathogenic mutations in relevant cell types. We introduced the pathogenic m.15150G > A mutation, showing functional effects on ATP production in human adult liver organoids. Similarly, we corrected the m.4291T > C mutation in fibroblasts derived from a patient with Gitelman-like syndrome and thereby improved mitochondrial function. In preparation for clinical mitochondrial base editing (mtBE), we demonstrated efficient delivery of the modified RNA (modRNA) mitochondrial base editors using lipid nanoparticles (LNPs), currently the most advanced non-viral *in vivo* delivery system for gene products. In summary, our research demonstrates the effectiveness of mtBE in primary adult human cells, leading to the recovery of mitochondrial function – two critical aspects for further development of mitochondrial gene therapy.

## Results

### Creation of m.15150G > A in patient-derived organoids

As the liver is a high-energy demanding organ affected by mitochondrial dysfunction, we set out to introduce a pathogenic mitochondrial mutation in human primary adult liver stem cell-derived organoids. We selected the m.15150G > A mutation in *MT-CYB* since the required C > T mutation is creatable with DdCBE and symptoms occur even at relatively low heteroplasmy levels [15,16]. Furthermore, this nonsense mutation is likely to completely abrogate Cytochrome B protein function. While m.15150G > A has not yet been associated with liver disease, other pathogenic mutations in *MT-CYB* have [17]. For precise targeting of DdCBE to m.15150G, we designed two sets of Left- and Right-TALEs (Fig 1A). Validation of all four Left/Right construct combinations in HEK293T cells demonstrated high efficiency of G → A editing at m.15150 with each combination (Fig 1B). We observed some bystander editing of m.15154C and m.15155C within the spacing region, although at significantly lower efficiencies than on-target editing (S1A Fig). The editing specificity, defined by the ratio of on-target to bystander editing, was higher for combinations with the R2 constructs (Fig 1C). These results indicate L1R2 and L2R2 to be the best combinations, although the much more efficiently installed m.15150G > A nonsense mutation likely obscures any functional effects of the downstream bystander edits, as m.15154C > T is a silent mutation and m.15155C > T is another nonsense mutation. We obtained these results only after we had selected L1R1 for functional analysis and organoid editing (see below), so for reasons of uniformity and the likely inferior effects of the bystander edits, we continued with L1R1 in further analysis. Western blot for cytochrome B in HEK293T cells edited with L1R1 showed decreased cytochrome B protein levels (Figs 1D and S1B).

**Fig 1:**
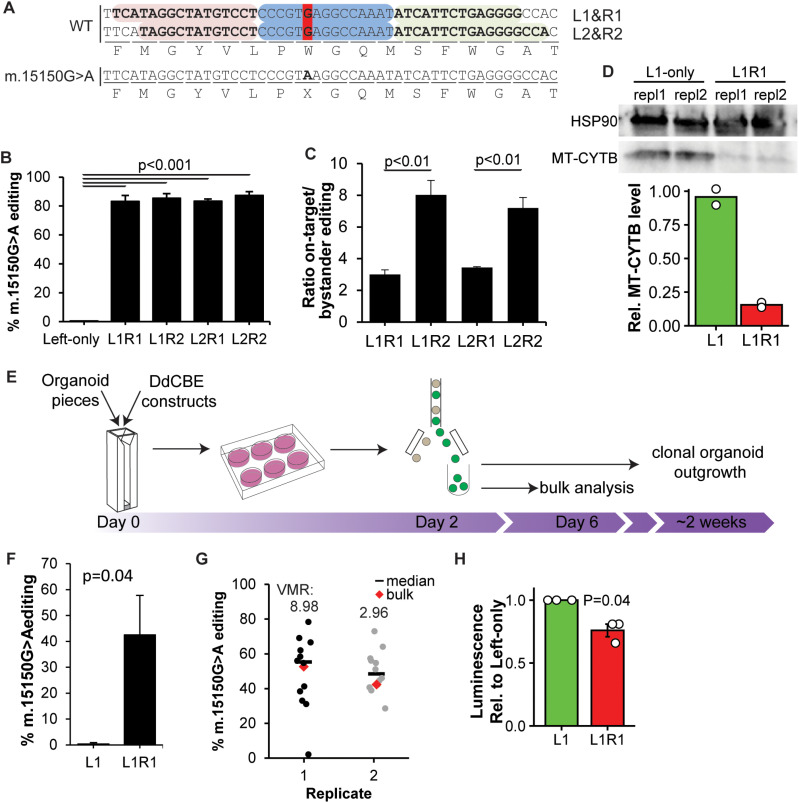
Creation of m.15150G > A using DdCBE in human liver organoids. (A) Design of two sets of Left (red) and Right (green) target sequences flanking the spacer region (blue) for TALE-guided editing in MT-CYB to create m.15150G > A (dark red). (B) Editing efficiencies of all four combinations of Left- and Right-DdCBE plasmids in HEK293T cells as determined by Illumina Next Generation Sequencing (NGS). L1- or L2-only were used as negative control. *N* = 3, one-way ANOVA with Tukey’s multiple comparisons post hoc test. (C) Ratio of on-target editing vs. bystander editing at any other base within the spacing region in HEK293T cells. *N* = 3, Welch’s *t* test. (D) Top: Reduced MT-CYTB protein expression in two technical replicates of m.15150G > A-edited HEK293T cells. Bottom: quantification of MT-CYTB bands relative to HSP90 as analyzed by densitometry. *N* = 2. (E) Overview of the transfection strategy of DdCBE for organoids. (F) Editing efficiencies of L1R1 constructs modRNA in liver organoids, Sanger sequencing *N* = 3, Welch’s *t* test. (G) Clonal lines of edi*t*ed organoids show high variability in mitochondrial heteroplasmy, indicating different editing efficiencies (Illumina NGS) per cell. Variance-to-mean ratios (VMRs) are displayed above each replicate (*N* = 12 clones for both replicates). (H) ATP levels decrease in organoids containing 24% m.15150G > A. *N* = 3, Paired *t* test. Raw data are provided in S1 Da*t*a.

We next set up a protocol for mitochondrial editing in organoids by electroporation and selection of transfected organoid cells through co-transfection with GFP by FACS (Fig 1E). Electroporating the machinery in organoids resulted in an average editing efficiency of 43% for m.15150G > A (Fig 1F). Since each cell contains hundreds to thousands of mtDNA molecules, editing efficiency may vary significantly between individual cells. To address this, we employed a single-cell approach to elucidate the intrinsic mtBE efficiency variability. Sequencing clonal organoids derived from single FACSed organoid cells revealed that editing efficiencies ranged from 0% to 80% (Fig 1G). The average variance-to-mean ratio (VMR) of two experiments is 5.96, indicating a relatively high dispersion. These results indicate that mtBE and clonal outgrowth of organoid lines could be a useful tool to study the effects of heteroplasmy on disease mechanisms.

Next, we examined whether introducing the m.15150G > A nonsense mutation in liver organoids led to reduced mitochondrial activity. After electroporating organoids with the editing machinery, we seeded FACSed GFP-high and GFP-low organoid cell populations on 2D culture plates. Four days after seeding, we measured ATP levels and found that liver cells with approximately 24% m.15150G > A mutation had 23% reduced ATP levels compared to control (Fig 1H).

### Correction of m.4291T > C in patient-derived fibroblasts

To investigate whether correcting a mitochondrial mutation in primary cells restores mitochondrial activity, we selected the m.4291T > C mutation (Fig 2A). This variant lies in the *MT-TI* gene, encoding the mitochondrial isoleucine tRNA, and affects the highly conserved uracil directly upstream of the anticodon [18,19]. We designed two sets of Left- and Right-TALEs (L1, L2, R1, R2) to guide DdCBE to m.4291C, introducing a mismatched but preferred thymine terminal Repeat Variable Diresidue (RVD) in L2 (Fig 2B). We adopted the organoid electroporation protocol for fibroblasts and seeded cells either bulk or single cell after FACS (Fig 2C). Testing the four DNA construct combinations on homoplasmic human skin fibroblasts revealed the L2R2 combination as the most efficient editor for restoring the *MT-TI* mutation (Fig 2D). No specific proximal bystander editing was detected (S2A Fig), making the L2R2 editor the most specific within the spacing region (Fig 2E). To compare gene correction efficiency between individual fibroblast cells, we derived clonal lines from single transfected fibroblasts, revealing significant variability in editing efficiency between individual fibroblasts (Fig 2F). As with liver organoids, the editing efficiency dispersion of skin fibroblasts was quite large, (average VMR: 6.8). To assess the stability of the edited mtDNA fraction in the cellular mtDNA pool, we sequenced edited fibroblasts (both clones and bulk lines) after more than 50 days of culture (Fig 2G). Interestingly, the m.4291T edited fraction increased with time in most fibroblast lines, reflecting the absence of a selective disadvantage of base edited mtDNA.

**Fig 2:**
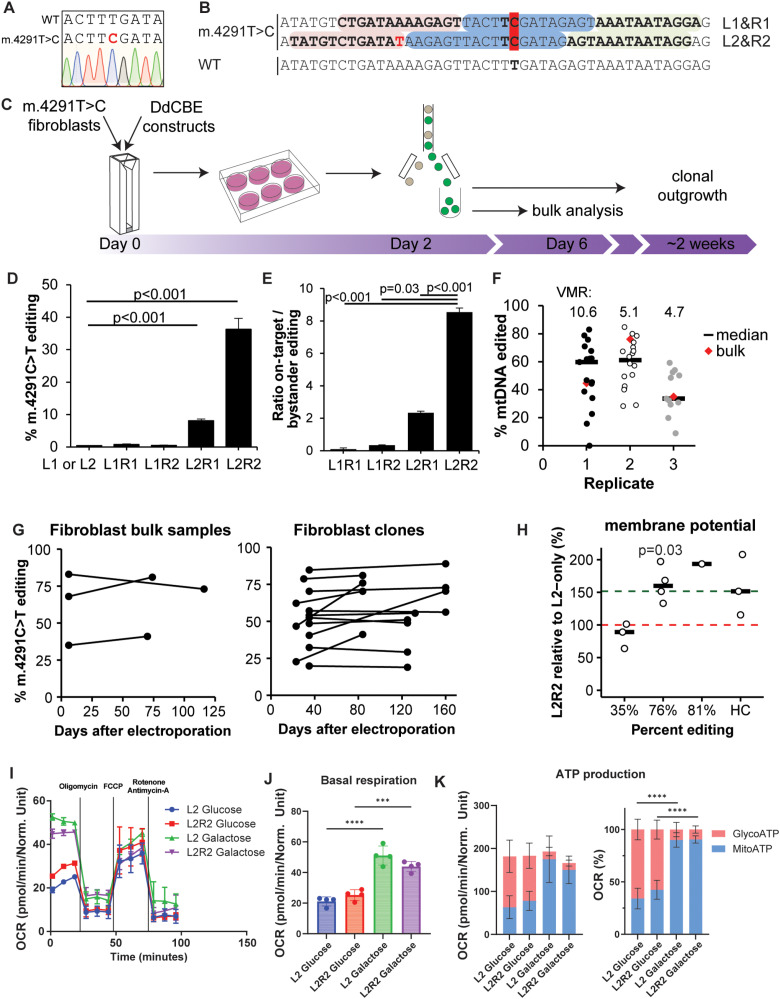
Correction of m.4291T > C in primary patient-derived fibroblasts. (A) The primary patient-derived fibroblast line is homoplasmic for the m.4291T > C mutation. (B) Design of two sets of Left (red) and Right (green) target sequences flanking the spacer region (blue) for TALE-guided editing in MT-TI to correct m.4291C > T (dark red). Mismatched T (red) in TALE target sequence L2. (C) Overview of the transfection strategy of DdCBE for primary skin fibroblasts. (D) Editing efficiencies of all four combinations Left- and Right-TALE-DdCBE plasmids in primary patient-derived fibroblasts as measured by amplicon sequencing (Illumina NGS). Transfection with Left-only DdCBE plasmid was used as negative control. *N* = 5 for L1/L2 and L2R2, *N* = 4 for L1R1 and L2R1, *N* = 3 for L1R2; one-way ANOVA with Tukey’s multiple comparisons post hoc test. (E) Ratio of on-target editing vs. bystander editing at any other base within the spacing region in fibroblast cells. One-way ANOVA with Tukey’s multiple comparisons post hoc test. (F) Clonal lines of edited primary patient-derived fibroblasts show high variability in mitochondrial heteroplasmy indicating different editing efficiencies per cell (Illumina NGS). *N* = 17, 18 and 11 for replicates 1–3, respectively. (G) Percentage of edited mDNA in primary patient-derived fibroblasts over time. This percentage slightly increased over time in most bulk and clonal cultures (Sanger sequencing). (H) Imaging flow cytometry analysis after TMRM-staining shows improved mitochondrial membrane potential in highly edited but not in lowly edited fibroblast lines (normalized to uncorrected control fibroblasts (red dashed line)). Green dashed line: average membrane potential of healthy control (HC) fibroblast lines from three individuals. *N* = 3 for 35%-edited line and HC, *N* = 4 for 76%-edited line, *N* = 1 for 81%-edited line; *t* test be*t*ween 76% edited and 35% edited lines. (I) Oxygen Consumption Rate (OCR) assessed by mitochondrial stress test on 81%-edited patient fibroblasts conditioned with either glucose or galactose, measured in basal conditions and after sequential injections of the following molecules modulating mitochondrial activity: oligomycin, FCCP, rotenone and antimycin-A. *N* = 5 technical replicates. (J) Basal respiration measured in 4 biological replicates. (K) ATP production in mitochondrial and glycolytic fractions measured in 4 biological replicates. *N* = 4; Error bars indicate Mean ± SD. **p < 0.01, ***p < 0.001, ****p < 0.0001; one-way ANOVA with Tukey’s multiple comparisons test. Raw data are provided in S1 Data.

Mutations in mitochondrial tRNA genes, such as *MT-TI*, have been reported to reduce mitochondrial membrane potential [20,21]. Correction of the m.4291T > C mutation is therefore expected to increase membrane potential of the patient-derived fibroblasts. Using image flow cytometry, we analyzed the functional effects of correction of this mutation in the context of heteroplasmy levels. Mitochondrial membrane potential, measured with TMRM staining, improved in patient-derived fibroblasts with 76% and 81% correction of the mutation (Fig 2H). Interestingly, this effect was not observed in fibroblasts with 35% correction of the mutation. We next performed Seahorse-based functional bioenergenics analysis on gene-corrected fibroblasts to gain more insight into mitochondrial activity after gene-correction. We observed a modest improvement in basal oxygen consumption rate (OCR) and ATP production when fibroblasts were conditioned in glucose medium but not in galactose medium which should make cells entirely dependent on mitochondrial respiration (Figs 2I and S3A and S3B). However, the observed improvements were not consistent between different analysis runs (Figs 2J and S3C).

While mitochondrial membrane potential is restored after correction of m.4291T > C, functional improvements of OCR are inconsistent, indicating that in corrected patient fibroblasts, mitochondrial functional restoration is modest at best.

### Mitochondrial base editing using modRNA and LNPs

Effective and safe delivery methods are essential for therapeutic application of mtBE. CRISPR-based editing tools delivered as modRNA encapsulated in LNPs have already been successfully applied to various *in vivo* models [22–25]. We therefore produced the DdCBE constructs targeting m.15150G and m.4291C as modRNA to investigate the potential for LNP-delivered modRNAs of the DdCBE to edit mtDNA in primary patient-derived cells. We first investigated the use of modRNA for m.15150G > A editing in organoids. Co-transfection with a GFP construct revealed a 17-fold higher transfection efficiency with modRNA compared to DNA plasmids (Fig 3A). Even after selection of transfected cells by GFP-FACS, mtBE efficiency was approximately four times higher in modRNA-transfected organoids than in DNA-transfected organoids at normal or double doses six days post transfection (Fig 3B). Bystander editing within the spacing region relative to on-target editing was similar between modRNA and DNA transfection (S4A Fig). To investigate the effects of using modRNA on cell death, we monitored cells for seven days post DNA and RNA transfection using electroporation. Transfection with DNA caused more cell death (>3 fold) in organoids than transfection with modRNA or without nucleotides for at least four days (Fig 3C). Next, we investigated the use of modRNA to correct the patient-derived fibroblasts. The transfection efficiency increased from 13.1% with DNA to 96.6% with modRNA encoding the DdCBE proteins and GFP (Fig 3D). With modRNA, the average efficiency of correction of the m.4291T > C was 59% (Fig 3E). These results demonstrate the potential for using modRNA as an *in vitro* delivery tool, which yields high efficiency while conserving viability. This may be particularly promising for difficult-to-maintain and difficult-to-transfect cells.

**Fig 3:**
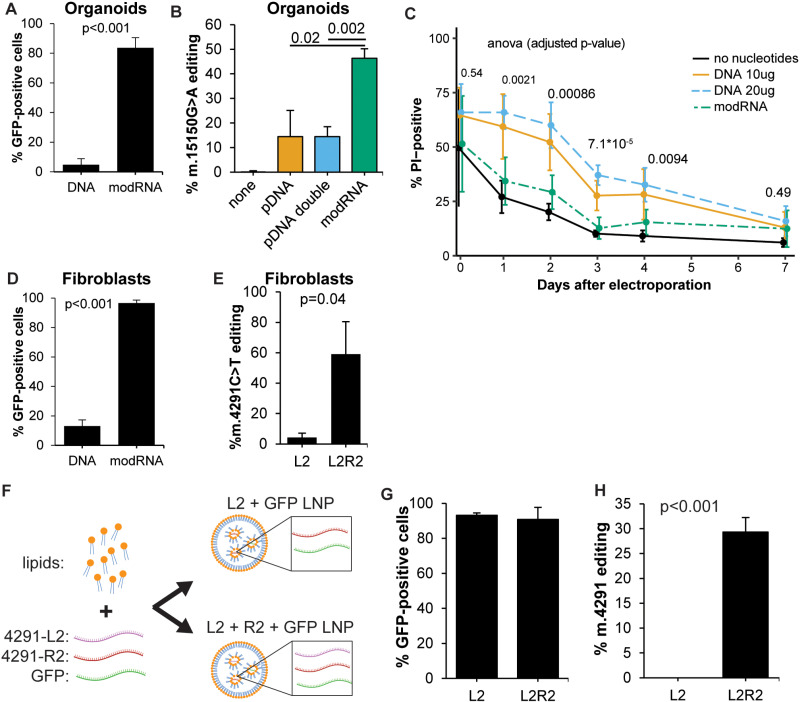
LNP-delivered modRNA encoding DdCBE efficiently corrects m.DNA mutations. **(A)** modRNA-encoded DdCBE delivery to liver organoids greatly increases transfection rates compared to DNA. *N* = 3 and *N* = 4 for DNA and modRNA-transfection, respectively. **(B)** modRNA-mediated DdCBE editing is more efficient than DNA-mediated editing to correct the m.15150G > A mutation in organoids as measured by amplicon sequencing (Illumina NGS). *N* = 4. (C) Cell viability as measured by propidium iodide (PI) positivity shows increased cell death for organoids electroporated with DNA but not for modRNA. *N* = 3. **(D)** modRNA-encoded DdCBE delivery to fibroblasts greatly increases transfection rates compared to DNA. *N* = 3. (E) Editing efficiency (Illumina NGS) of the m.4291T > C mutation six days after transfection with modRNA. *N* = 4. (F) Overview of LNP production. (G) Percentage of GFP-positive cells three days after LNP transfection. *N* = 3. (H) Efficiency of correction of m.4291T > C in patient fibroblasts (Sanger sequencing) using modRNA-DdCBE delivered with LNPs. *N* = 3. All *P*-values were calculated with Student *t* test, or ANOVA (C). Raw da*t*a are provided in S1 Data.

To test the compatibility of DdCBE-mediated mtBE and gene delivery by LNPs, we packaged the modRNA-encoded DdCBE machinery targeting m.4291T > C along with *GFP*-modRNA in LNPs (Fig 3F). Three days after treating m.4291T > C fibroblasts with these LNPs, the transfection efficiency as measured by flow cytometry was more than 90% for both L2-only and L2/R2 conditions (Figs 3G and S4B and S4C). The efficiency of this delivery method was therefore comparable to modRNA-lipofection with a commercial kit (compare with Fig 3D). Importantly, delivery of 1 µg modRNA-encoded DdCBE with LNPs to 12,500 primary patient-derived fibroblasts resulted in on average 28% editing. This highlights the potential of this approach for therapeutic gene editing (Figs 3H and S4D).

### Analysis of off-target editing in corrected patient fibroblasts

We searched for the presence of off-target editing in the gene-corrected fibroblasts. We selected nine off-target sites (all in the nuclear genome) with high homology to the L2- and R2-TALE target sequences (S2 Data). NGS amplicon sequencing of these sites revealed small percentages of C•G > T•A substitutions (up to 0.2% of sequencing reads) within the spacing regions (Fig 4A). However, in three different replicates, none of these substitutions were consistently higher in abundance in gene-edited fibroblasts than in uncorrected L2-only control fibroblasts (Fig 4B). This suggests that The DdCBE-m.4291-L2R2 base editor had no significant off-target editing activity at least at these nine highly homologous off-target sites. We further looked for proximal bystander editing beyond the spacing region within the on-target amplicon region in the mitochondrial genome (S5 Fig). We found two positions (m.4333 and m.4354, both present in *MT-TQ*) at which at least one of the L2R2 replicates had >1% C•G > T•A substitution abundance that was more than 2-fold higher than in the L2-only condition, which suggests proximal bystander editing by DdCBE-m.4291-L2R2. Analysis of the whole mitochondrial genome for off-target editing did not show again these particular substitutions (Fig 4C). These substitutions could therefore be artifacts of the PCR amplification process prior to NGS. We instead observed >1% C•G > T•A substitutions at other locations specifically in L2R2-conditions which we regarded as off-target editing. The number of locations with off-target editing seemed to correlate with on-target editing efficiency (40, 0, and 6 locations for replicates 1, 2 and 3 (81%, 18% and 45% editing, respectively.

**Fig 4:**
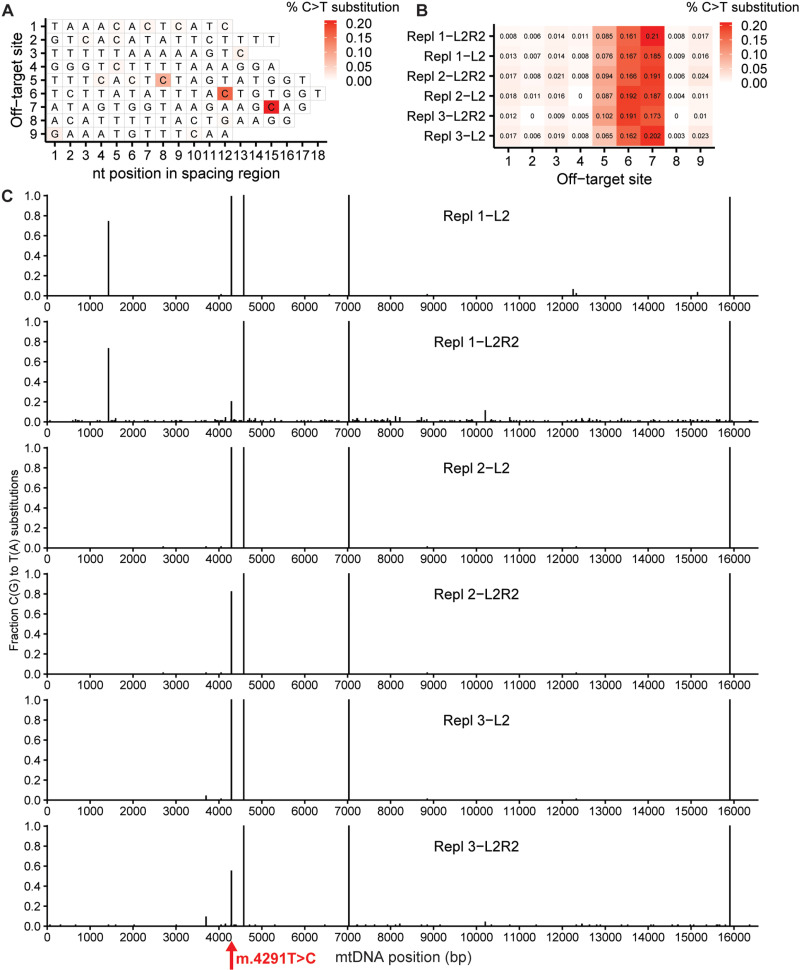
Off-target editing analysis in m.4291C-corrected fibroblasts. **(A)** Heatmap of C•G > T•A substitution abundances (percentage of reads) within spacing regions at nine different off-target sites with homology to the m.4291-L2- and m.4291-R2-TALE target sequences in 78%-edited patient fibroblasts. **(B)** Heatmap of C•G > T•A substitution abundances for the most abundant substituted cytosines (highlighted in **A**) within the nine spacing regions in three different gene-corrected fibroblasts and uncorrected L2-only control fibroblasts. Replicates 1–3 had 78%, 20% and 46% on-target editing, respectively. **(C)** C•G > T•A substitution abundances as fraction of reads across the mitochondrial genome for all six fibroblast lines. The on target editing at m.4291 is depicted as T > C substitution and therefore lower in L2R2-conditions. The presence of homoplasmic substitutions in all six lines indicates naturally occurring variants. Replicate 1 contains in both L2-only and L2R2 a heteroplasmic m.1428G > A variant. Data in **(A)** and **(B)** produced with Illumina NGS, data in **(C)** produced with Illumina NovaSeq. Raw data are provided in S1 Data.

In summary, we observed no off-target editing in nine selected sequence-dependent off-target sites in the nuclear genome, while mitochondrial off-target editing seemed to correlate with on-target editing, as expected, though the sample size is small.

## Discussion

This study demonstrates the efficient creation and correction of mitochondrial DNA-mutations in primary, patient-derived fibroblasts and human adult liver stem cell-derived organoids. Furthermore, we delivered DdCBE-modRNA via LNPs to patient-derived fibroblasts to correct the m.4291T > C mutation, demonstrating the compatibility of mtBE with modRNA-LNPs. Correction of m.4291T > C functionally restored mitochondrial membrane potential, but did not significantly improve mitochondrial ATP production or oxidative respiration in corrected fibroblasts. This finding warrants further functional studies in the context of mitochondrial base editing to also assess the consequences of mitochondrial off target editing for instance. Yet the potential therapeutic value of this precise gene editing technique for mitochondrial diseases is supported by the recently reported improvement in oxidative respiration following correction of a pathogenic mitochondrial variant in iPSCs [26]. Our study provides evidence for the potential of DdCBE to functionally correct a mitochondrial pathogenic variant in primary patient-derived cells. Importantly, we observed no overt loss in the fraction of corrected mitochondrial DNA within bulk and clonal populations over several weeks of culture. This indicates that the edited mtDNA remains stable and functional and that gene editing does not confer a selective disadvantage over time, at least not for this particular mutation. We rather observed a slight net advantage of the edited mtDNA fraction among all clones, which indicates the potential long-term viability and effectiveness of this mitochondrial DNA correction approach. If there were a selective advantage associated with edited mitochondrial DNA (mtDNA), it would be expected that fibroblast clones with low edited mtDNA fractions would increase this fraction over time to a greater extent than clones with already high edited mtDNA fractions. However, this was only observed for a subset of clones. It remains to be investigated to what extent mtBE induces mitochondrial silencing [27], and how newly induced edits are cellularly inherited during cell division [28,29]. Of note, for both organoids and fibroblasts, there was high variability in editing efficiency among the different clones, suggesting that the efficiency of mtBE is dependent on specific cell states, such as the cell cycle stage.

To prepare for future *in vivo* mitochondrial gene editing, we examined delivery strategies for gene editors that are currently evolving into curative gene correction therapies for nuclear genetic disorders [30–33]. So far, *in vivo* application of mtBE has been limited to AAV technology [13,14]. However, viral delivery is associated with toxicity, immunological reactions and undesired editing effects caused by long-term expression of the gene-editors [34]. These unwanted effects of viral delivery were addressed for nuclear DNA-editing by delivering base editors as modRNA encapsulated in LNPs [22,23]. In this study we investigated the delivery of DdCBE as modRNA packaged in LNPs. The appreciable levels ( approximately 30%) of the m.4291C correction in primary human fibroblasts highlight the potential of efficient *in vivo* delivery of mitochondrial gene editing. However, LNPs naturally accumulate in the liver after intravenous administration where they allow for high modRNA-payload delivery [35]. Although this is promising for mitochondrial liver disease, further research is needed to target other organs affected by mitochondrial diseases. In this context, it is noteworthy that the LNP-field is rapidly advancing toward active organ-targeting strategies [36,37]. Our results with LNP delivery of DdCBE modRNA indicate that we can build on the recent advancements in the delivery field of nuclear gene editors for targeted organ delivery. It will be important to investigate viral and non-viral delivery strategies in relevant animal models to determine whether mtBE can achieve sufficient editing efficiency for clinical application in the treatment of various different mitochondrial diseases.

Our studies also demonstrate the potential of DdCBE to generate mitochondrial *in vitro* models. Firstly, we show that modRNA is a potent means of editing relatively difficult-to-transfect cell types such as primary fibroblasts and organoids by improving transfection efficiency, DdCBE editing efficiency, long-term stability, and cell viability. Secondly, clonal lines with different levels of heteroplasmy were generated from organoids and fibroblasts. These provide a powerful source for studying disease mechanisms at different levels of heteroplasmy to attain unique insights in the effects of editing efficiency on disease phenotype.

Our studies have shown that the effectiveness of editing outcomes achieved with DdCBE largely depends on the design of the editing machinery. Careful optimization of the DdCBE mtBE system is therefore essential to minimize bystander effects and ensure effective editing outcomes. Recent advancements have significantly improved the flexibility and precision of mtBE [2–5,11,12], and suggest that technological advancements for mitochondrial gene editing will follow a similar trajectory to CRISPR-based nuclear DNA editing [38]. To realize the full potential of mtBE as a gene-correction tool, further improvements and specifically diversifications of the mtBE machinery are necessary to expand applicability to a broader spectrum of mutations.

One important aspect to consider is the occurrence of off-target effects in the mitochondrial genome which may affect mitochondrial function. We have used Whole Genome Sequencing (WGS) by long-range PCR to assess off-targets in the mitochondrial genome, as have various other studies [2,6,14,26]. A downside of this method, however, is the lack of sequencing-depth to achieve sufficient sensitivity to counteract the dilutive effect of the cellular abundance of the mitochondrial genome. An interesting alternative method uses nanopore sequencing-mediated long read WGS to achieve molecule level resolution of mutations [39].

In conclusion, our results demonstrate that mtBE is a promising and precise gene editing tool for creating and correcting mitochondrial mutations in primary patient-derived cell types, and is well compatible with modRNA and LNP delivery. While there are challenges and areas for further investigation, the potential of mtBE in disease modeling and potential therapeutic interventions makes it a promising avenue for future research and development in mitochondrial medicine.

## Materials and methods

### Study approval and human subjects

The study was approved by the responsible local ethics committees (Institutional review board of the University Medical Center Utrecht (STEM: 10-402/K) and Arnhem-Nijmegen (2019–5749)) [19]. All patient biopsies were used after written informed consent.

### Cloning

Left and Right TALE sequences were ordered as synthesized DNA (Genscript) and cloned into pDdCBE plasmids with either G1397 DddA-C or G1397 DddA-N constructs (Addgene) by PstI/BamHI digestion followed by T4 ligation. All Left and Right TALE target sequences and translated products are listed in S2 Data. To improve ease-of-cloning of target genes into the backbone, we modified the pcDNA3.3 by introducing BbsI restriction sites between the 5′ UTR and 3′ UTR using in-fusion cloning (Takara). DdCBE constructs were cloned into the pcDNA4 plasmid by digestion of the destination vector with BbsI, PCR of the DdCBE constructs and subsequent in-fusion cloning.

### ModRNA

ModRNA production was performed as previously described [40]. In short, modRNA production plasmids were linearized using SpeI-HF (NEB). Next, PCR was performed with Q5 High-Fidelity 2X Master Mix on the linearized modRNA production plasmids using the Xu-F1 and Xu-T120 primers containing a poly-A sequence as used by Mandal and Rossi [40]. *In vitro* transcription was performed using the MEGAscript T7 Transcription Kit (ThermoFisher Scientific) according to manufacturer’s protocols using 25–50 ng/µl PCR product, N1-Methylpseudouridine-5′-Triphosphate (N-1081,TriLink) and 3′-O-Me-m7G(5′)ppp(5′)G RNA Cap Structure Analog (S1411, NEB). Then the modRNA was purified with the RNeasy Mini Kit (Qiagen) and the RNA concentration was measured by fluorometric quantification (Qubit, ThermoFisher Scientific).

### Preparation of lipid nanoparticles

LNPs were produced by microfluidics mixing in a NanoAssemblr Benchtop device (Precision Nanosystems, Vancouver, Canada). Before production, lipids were diluted to a total lipid concentration of 10 mM in pure ethanol while mRNA was diluted in a 100 mM acetate buffer (pH4). LNPs were produced with a wt/wt ratio (ionizable lipid/mRNA) of 12:1 at a total flow rate of 9 mL/min, flow rate ratio of 3:1 (aqueous:lipid phase) at room temperature. The composition of the mRNA phase was Left-/Right-/GFP at a weight ratio of 5/5/2, respectively. Lipid phase was composed of SM-102 (Cayman Chemicals, Ann Arbor, MI, USA)/Cholesterol (Sigma Aldrich, Saint Louis, MO, USA)/DSPC (Lipoid, Ludwigshafen am Rhein, Germany)/DMG-PEG2000 (Avanti polar lipids, Inc, Alabaster, AL, USA). at a molar ratio of 50/38.5/10/1.5, respectively. LNPs were then dialyzed overnight into TBS using slide-a-Lyzer dialysis cassettes G2 20 kDa (ThermoFisher). LNPs were characterized by size, zeta potential and encapsulation efficiency.

### Organoid culture

Liver organoids were plated in Matrigel (Corning) and maintained in human liver expansion medium (hL-EM), consisting of AdDMEM/F12 (Gibco) supplemented with, GlutaMAX (1×, Gibco), HEPES (1×, Gibco), PenStrep (1×, Gibco), 2% B27 without vitamin A (Gibco), 1.25 mM *N*-Acetylcysteine (Sigma), 10 mM Nicotinamide (Sigma), 10 nM gastrin (Sigma), 10% RSPO1 conditioned media (homemade), 50 ng/ml EGF (Peprotech), 100 ng/ml FGF10 (Peprotech), 25 ng/ml HGF (Peprotech), 5 mM A83-01 (Tocris), and 10 mM FSK (Tocris). The medium was changed every 2–4 days and organoids were passaged 1:4 to 1:8 each week. After thawing, organoids were passaged at least once before electroporation.

### Cell culture

Fibroblasts were maintained and split every 7 days in standard medium, consisting of F-12 Nut Mix (Ham) (Gibco), 10% FBS (Gibco), and PenStrep (1×, Gibco). HEK293T cells were maintained and split every 4–5 days in standard medium, consisting of DMEM + GlutaMAX (1×, Gibco), 10% FBS (Gibco), and PenStrep (1x, Gibco).

### Transfection of HEK293T cells

HEK293T cells were plated in standard medium at a density of 80,000 cells per well in a 24 wells plate 1 day prior to transfection. HEK293T cells were transfected with the following for DNA: 1 µg Left-plasmid, 1 µg Right-plasmid and 200 ng GFP plasmid in a mix of 50 µl OptiMEM and 2 µl Lipofectamine 2000 (Thermo Fisher) for each well. For modRNA, HEK293T cells were transfected with 500 ng Left-transcript, 500 ng Right-transcript and 100 ng GFP transcript in a mix of 50 µl OptiMEM and 1 µl Lipofectamine 2000 for each well unless stated otherwise.

### Organoid and fibroblasts electroporation

For organoid electroporation, organoids were grown under standard culture conditions. Organoids (one 6-well per electroporation reaction) were briefly dissociated using TrypLE (Gibco) for 4–5 min at 37°C, after which mechanical disruption was applied through pipetting. The resulting small organoid pieces were washed once using Advanced DMEM/F12, resuspended in 80 µl OptiMEM containing Y-27632 (10 µM). For fibroblast electroporation, cells were grown and harvested under standard culture conditions. 500,000–1,000,000 cells were used per transfection and resuspended in 80 µl OptiMEM per transfection. For both fibroblasts and organoids, 20 µl DNA or modRNA mixture was added. For DNA transfection, the mixture contained 9 µg Left-, 9 µg Right- and 2 µg GFP-plasmid. For RNA transfection, the mixture contained 5 µg Left-, 5 µg Right- and 2 µg GFP-transcript unless stated otherwise. The cell/nucleic acid mixture was transferred to an electroporation cuvette and electroporated using a NEPA21 electroporator (NEPA GENE) with 2 × poring pulse (voltage: 175 V, length: 5 ms, interval: 50 ms, polarity: + , decay rate: 10%) and 5 × transfer pulse (voltage: 20 V, length: 50 ms, interval: 50 ms, polarity + /-, decay rate: 40%), as previously described [41]. Cells were removed from the cuvette and transferred into 500 µl OptiMEM containing Y-27632 (10 µM). After 20 min, organoids were plated in 120 µl Matrigel per condition, while fibroblasts were seeded in one well of a 6 wells plate per condition in 2 ml medium. Upon polymerization of the Matrigel, hL-EM was added containing Y-27632 (10 µM).

### FACS and organoid reconstitution

Organoids and cell lines were harvested and dissociated to single cells using TrypLE (Gibco) or Trypsin (Gibco), respectively, after which cells were resuspended in FACS buffer (phosphate-buffered saline with 2 mM ethylenediaminetetraacetic acid, 0.5% bovine serum albumin) and 1ug/mL DAPI (Sigma-Aldrich D9542). Prior to FACS, cells were filtered through a 5 mL Falcon polystyrene test tube (Corning). Sorting was performed on the FACS FUSION (BD) using FACS Diva software (BD). Live cells were sorted based on lack of DAPI signal, then sorted for successful transfection based on GFP signal, collected in tubes containing culture medium and plated accordingly. For clonal outgrowth of fibroblasts, single cell fibroblasts were sorted in 96 wells plates. For clonal outgrowth of organoids, single cells were seeded sparsely in Matrigel, and after around two weeks, single organoids were picked, sequenced and expanded.

### Sanger sequencing

6 days after initial transfection of the editing machinery, cells were harvested using the Quick-DNA microprep kit (Zymogen) according to the manufacturer’s protocols. PCR was performed on the genomic region of interest using the Q5 High-Fidelity 2X Master Mix and purified using the NucleoSpin Gel and PCR Clean-up kit (Macherey-Nagel) according to manufacturer instructions. The PCR product was sent for Sanger sequencing to EZSeq Macrogen Europe. All PCR and sequencing primers used are listed in S2 Data. The online tool EditR was used to determine editing efficiency of on-target and bystander edits [42].

### NGS sequencing

Genomic sites of interest were amplified from genomic DNA samples and sequenced on an Illumina iSeq 100 as previously described [43]. In short, PCR primers containing Illumina forward and reverse adapters (S2 Data) were used in a first amplification reaction (PCR1) of 25 µl using Q5 polymerase (NEB) to amplify the genomic region of interest. In a second round of PCR (PCR2, 25 µl), 1 µl of each PCR1 was barcoded with unique Truseq DNA Index primers (Illumina) and isolated from gel. DNA concentration was measured by fluorometric quantification (Qubit, ThermoFisher Scientific) and sequenced on an Illumina iSeq 100 instrument according to the manufacturer’s protocols to create 2 × 150 bp paired-end reads. The resulting FASTQ files were analyzed with the online tool RGEN BE-Analyzer, using the unedited sequence as the reference sequence and the spacing region as the target DNA sequence [44]. Similarly, C•G > T•A substitutions were identified within the amplicon using RGEN BE-analyzer.

### Whole mitochondrial genome sequencing

Long-range PCR was performed to amplify the entire mitochondrial genome with two sets of primers (see S2 Data) and PRIMESTAR GXL DNA polymerase (Takara). Twenty to 40 ng of isolated genomic DNA was used as template. The following PCR program was performed: 94°C for 1 min, then 30 cycles of 98°C for 30 s, 60°C for 30 s, and 72°C for 9 min followed by a final extension at 72°C for 20 min. Approximately 8.5 kb PCR products were excised from agarose gel for purification. For each condition, both PCR products from the two sets of primers were pooled from which the library was created using the TruSeq Nano DNA kit (Illumina). 150 bp paired-end sequencing was done using NovaSeq X at 2 Gb per sample. GRCh38.p14 (GCF_000001405.40) was used as reference genome and was indexed using samtools (v1.13). Positions with conversion rates >0.1% among all bases in the mitochondrial genome were identified using reditools/analyze.py script from REDItools (v3.3) with parameters set to -mrl 30 -q 30 -e -d -l 1 -me 1 -s.

### Nuclear off-target editing analysis

For sequence-dependent off-target editing analysis, genomic sites that were homologous or near homologous to the 4291-L2 and 4291-R2 TALE target sequences were selected. For this, the TALE RVD sequences were submitted to the online tool Cornell university TAL effector nucleotide targeter 2.0 [45]. Potential Left- and Right-targets were selected by having individual scores of >4. Left- and Right-target pairs were identified by their proximity to be between 10 and 18 nt (size limits of the spacing region). Thus, a shortlist was generated for which amplicon primers were designed; those sites for which no primers could be designed were removed from the list. The top nine sites of this list were selected for NGS sequencing.

### Organoid viability assay after modified RNA electroporation

Human adult liver organoids were electroporated with DNA, RNA or without nucleotides and plated in 24 wells of a 96 wells plate. At every time point, 3 wells were incubated with 10 µg/ml Hoechst 33342 (ThermoFisher, cat.nr) and 0.1 mg/ml propidium iodide (PI) (Eurogentec, AS-83215) in the dark for 20 min before imaging. Wells were screened using the THUNDER microscope (Leica microsystems) and live/dead cells were quantified using QuPath [46]. Cells were annotated using Hoechst signal, and the fraction of dead cells determined by PI-signal. The same PI-signal threshold for determining PI-positivity was used for all conditions. Three technical replicates were analyzed per condition and averaged for each biological replicate.

### ATPlite assay

Organoids were electroporated with DdCBE-15150-L1, DdCBE-15150-R1 and GFP modRNA (DdCBE-15150-R1 was omitted in the negative control condition). After 2 days of electroporation, 4,000 GFP-positive organoid cells were FACSed and seeded in two 96-wells to grow as 2D monoculture. 6 days after electroporation, the cells were first stained with Hoechst and imaged to count the number of cells, then ATP levels were determined with the ATPlite kit (Perkin Elmer, 6016943). Luminescence signal was normalized to the number of cells in corresponding wells.

### Western blot

HEK293T cells were transfected with DdCBE-15150-L1, DdCBE-15150-R1 and GFP plasmids (DdCBE-15150-R1 was omitted in the negative control condition). Two days after transfection, GFP-positive cells were selected with FACS and allowed to grow for four more days before they were lysed with Laemmli buffer with protease inhibitors (Roche, 11836170001; Thermo Fisher, 87786). Forty micrograms protein per sample was loaded on a 4%–15% Mini-PROTEAN TGX Stain-Free Protein gel (Bio-Rad, 4568085), separated by electrophoresis, and transferred to Immobilon-P membranes (Millipore). These membranes were blocked in TRIS-buffered saline with 0.1% Tween-20 and 5% bovine serum albumin for 1 h and subsequently incubated overnight at 4°C with primary antibodies in 0.5% bovine serum albumin–TRIS-buffered saline with 0.1% Tween-20. After washing, membranes were incubated with horseradish-peroxidase–conjugated secondary antibodies. Enhanced chemiluminescence western blotting detection reagents (SuperSignal West Dura Extended Duration Substrate, Thermo Fisher, 34075) were used to detect immunoblotted proteins. The following primary antibodies were used: anti-MT-CYTB (Abcam, AB219823), anti-HSP90 (kind gift from Prof. Jeffrey Beekman).

### Imaging flow cytometry

Cells were plated in 6-well plates to reach 70%–80% confluency at the day of the assay. At the day of the assay, cells were incubated with the following compounds TMRM (30 nM, Sigma–Aldrich), NAO (50 nM, Enzo Life Sciences) at 37 degrees in HBSS for 40 min. FCCP (3 μM, TargetMol) was added in the last 5 min of incubation. Cells were subsequently washed once with PBS0 and incubated with TrypLE (Gibco) for 2 min. Cells were harvested using 1 mL 10% dialyzed FBS (Gibco) in PBS without calcium or magnesium, spun down, and immediately visualized using ImageStream flow cytometer. Since lipophilic cations like TMRM are extruded by MultiDrug Resistance transporters, all experiments were performed within 30 min after the staining procedure.

### Mitochondrial stress test

Oxygen consumption rates (OCR) were measured using a Seahorse XFe24 Analyzer (Agilent Technologies). One day prior to analysis, fibroblasts were seeded at 7,500 cells per well in a Seahorse 24-wells plate (Agilent Technologies, 102342-100) that was precoated with Poly-D-Lysine (Thermo Fisher, 16021412). The fibroblasts were kept overnight on either 4.5 g/L D-glucose or 1.8 g/L galactose in DMEM SILAC medium (Thermo Fisher, 15318005) supplemented with 10% FBS (Gibco), and PenStrep (1×, Gibco). One day after seeding, medium was changed to Agilent XF DMEM (103680-100, Agilent) supplemented with 4.5 g/L glucose (Gibco, A2494001), or 1.8 g/L galactose (Sigma–Aldrich, G0750), 2mM L-Glutamine (Gibco, 25030081) and 1 mM Sodium pyruvate (Gibco, 11360070), and incubated 1 h at 37°C in the absence of CO_2_. Plates were analyzed with the Seahorse XFe24 Analyzer as previously described [47], but using 1 μM Oligomycin (Merck, O4876-5MG), 1.5 μM FCCP (MedChemExpress, HY-100410), 1 μM Rotenone (MedChemExpress, HY-B1756) and 1 μM Antimycin-A (Sigma, A8674) while D-glucose and galactose conditions were maintained. With the last injection we included Hoechst 33,342 (1:1000; Thermo Fisher) for subsequent cell count which was used for normalization. Plates were scanned with the Leica Thunder microscope. OCR for Basal respiration and ATP production were calculated using the Seahorse Wave software (version 2.6; Agilent). ATP production rates, consisting of mitoATP and glycoATP values were calculated using published formulas [48,49].

## Supporting information

S1 Fig **(A)** Percent G > A or C > T editing (Illumina NGS) in the m.15150 spacing region in HEK293T for all four different Left/Right construct combinations. *N* = 3. **(B)** The complete image of western blot in Fig 1D. Raw data are provided in S1 Data.
(PDF)

S2 Fig **(A)** Percent G > A or C > T editing in the m.4291 spacing region in m.4291T > C patient fibroblasts for all four different Left/Right construct combinations. *N* = 5 for L1/L2 and L2R2, *N* = 4 for L1R1 and L2R1, *N* = 3 for L1R2; Illumina NGS, **(B)** Various cellular characteristics in m.4291C-corrected fibroblasts at multiple different degrees of correction, as measured by ImageStream flow cytometry, normalized to uncorrected control fibroblasts (gray dashed line). HC: control skin fibroblasts derived from healthy individuals. Raw data are provided in S1 Data.
(PDF)

S3 Fig Oxygen Consumption Rate (OCR) assessed by mitochondrial stress test using the Seahorse XFe24 Analyzer on primary patient fibroblasts conditioned with either glucose or galactose. OCR was measured in basal conditions and after sequential injections of the following molecules modulating mitochondrial activity: oligomycin, FCCP, rotenone and antimycin-A. **(A)** Basal respiration measurements from the mito-stress test displayed in Fig 2I (*N* = 5 technical replicates). **(B)** ATP production as absolute and relative (%) values from the mito-stress test displayed in Fig 2I. **(C)** OCR plot displaying the average of four biological replicates. L2R2 cells have 81% correction editing. *N* = 4; Error bars indicate Mean ± SD. *p < 0.05, **p < 0.01, ***p < 0.001, ****p < 0.0001; one-way ANOVA with Tukey’s multiple comparisons test. Raw data are provided in S1 Data.
(PDF)

S4 Fig **(A)** Percent G > A or C > T editing in the m.15150 spacing region in liver organoids six days after electroporation with modRNA (*N* = 4), DNA plasmids (*N* = 3) or double DNA plasmid (*N* = 2) amounts for L1-only or L1R1-combination constructs. Illumina NGS. **(B)** Cell viability (percent of DAPI-negative cell-sized events) three days after LNP-transfection of fibroblasts with different amounts of modRNA encoding DdCBE-4291-L2R2. **(C)** Transfection efficiency (percent of GFP-positive live cells) of conditions in **(B)**. **(D)** m.4291C > T editing efficiencies six days after LNP-transfection. *N* = 3 for 1 μg-condition only. Sanger sequencing. Raw data are provided in S1 Data.
(PDF)

S5 Fig C•G > T•A substitution abundances as fraction of reads for the same six fibroblast lines along the entire on-target amplicon. Two positions with likely bystander editing are indicated with red arrows. All data produced with Illumina NGS. Raw data are provided in S1 Data.
(PDF)

S1 Raw Images Original raw blot images used for Figs 1D and S1B. **(A)** The complete immunoblot image captured with a ChemiDoc Bio imaging system after anti-HS90 (top half) and anti-CYTB staining (bottom right portion). The bottom left portion of this blot was stained with a different antibody and not used for this study. Lanes not included in the final figures are marked with an “X” above the lane label. **(B)** Colorimetric image of the same blot to indicate the molecular weight marker bands.
(TIF)

S1 Data Original raw data used to generate Figs 1–4 and S1–S5. (XLSX)

S2 Data Tables containing the TALE protein sequences of the DdCBE-4291 and DdCBE-15150 editors, the primers used for sequencing and information on the nine sequence-dependent off-target sites. (XLSX)

## Abbreviations

AAV: adeno-associated virus
mtBE: mitochondrial base editing
LNPs: lipid nanoparticles
OCR: oxygen consumption rate
RVD: repeat variable diresidue
TALEs: transcription activator-like effectors
VMRs: variance-to-mean ratios
WGS: whole genome sequencing
modRNA: modified RNA
TMRM: tetramethylrhodamine methyl ester
HC: healthy control
DdCBE: deaminase toxin A-derived cytosine base editor
