# Supplementary figures and images for: Correction of pathogenic mitochondrial DNA in patient-derived disease models using mitochondrial base editors
Source: PLoS Biol. 2025 Jun 24;23(6):e3003207. doi: 10.1371/journal.pbio.3003207 (PMC12186987; doi:10.1371/journal.pbio.3003207)

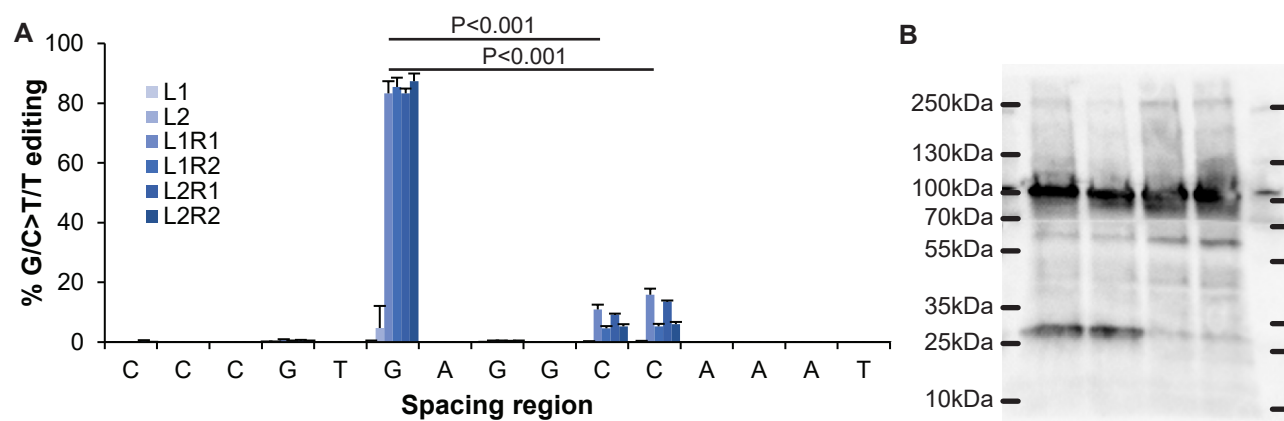

Figure S1

Supplement: S1 Fig — (A) Percent G > A or C > T editing (Illumina NGS) in the m.15150 spacing region in HEK293T for all four different Left/Right construct combinations. N = 3. (B) The complete image of western blot in Fig 1D. Raw data are provided in S1 Data. (PDF) [file pbio.3003207.s001.pdf]

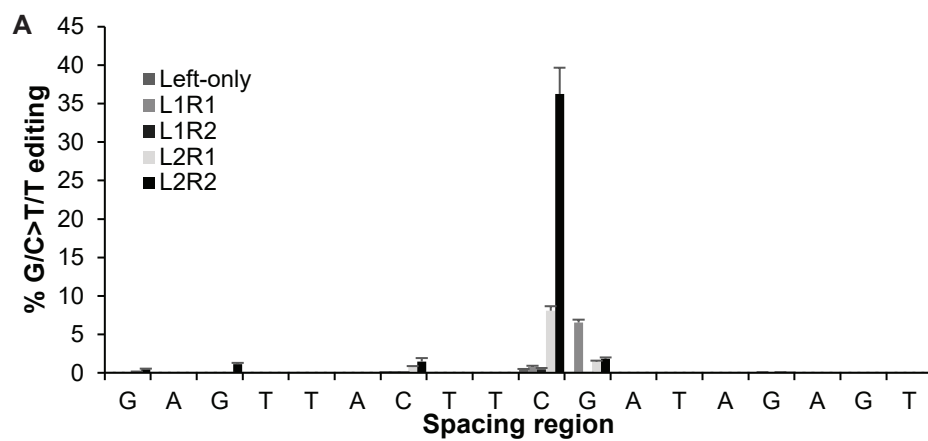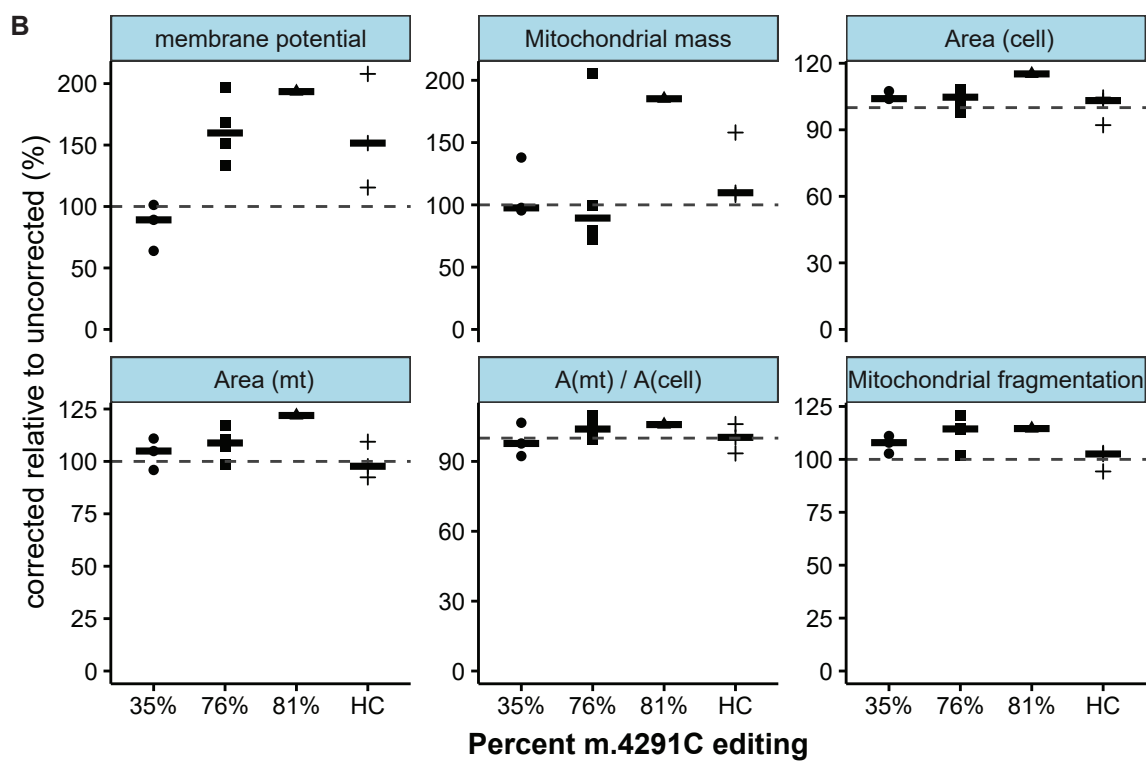

Supplement: S2 Fig — (A) Percent G > A or C > T editing in the m.4291 spacing region in m.4291T > C patient fibroblasts for all four different Left/Right construct combinations. N = 5 for L1/L2 and L2R2, N = 4 for L1R1 and L2R1, N = 3 for L1R2; Illumina NGS, (B) Various cellular characteristics in m.4291C-corrected fibroblasts at multiple different degrees of correction, as measured by ImageStream flow cytometry, normalized to uncorrected control fibroblasts (gray dashed line). HC: control skin fibroblasts derived from healthy individuals. Raw data are provided in S1 Data. (PDF) [file pbio.3003207.s002.pdf]

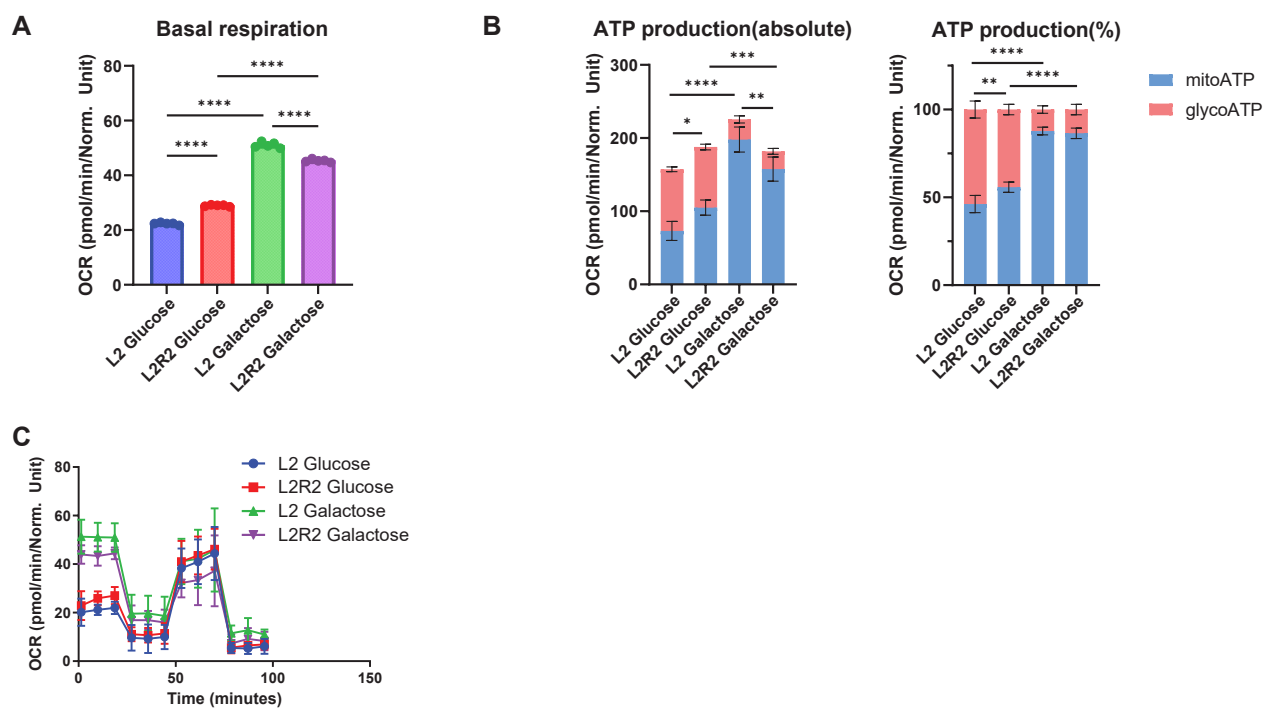

Figure S3

Supplement: S3 Fig — OCR was measured in basal conditions and after sequential injections of the following molecules modulating mitochondrial activity: oligomycin, FCCP, rotenone and antimycin-A. (A) Basal respiration measurements from the mito-stress test displayed in Fig 2I (N = 5 technical replicates). (B) ATP production as absolute and relative (%) values from the mito-stress test displayed in Fig 2I. (C) OCR plot displaying the average of four biological replicates. L2R2 cells have 81% correction editing. N = 4; Error bars indicate Mean ± SD. *p < 0.05, **p < 0.01, ***p < 0.001, ****p < 0.0001; one-way ANOVA with Tukey’s multiple comparisons test. Raw data are provided in S1 Data. (PDF) [file pbio.3003207.s003.pdf]

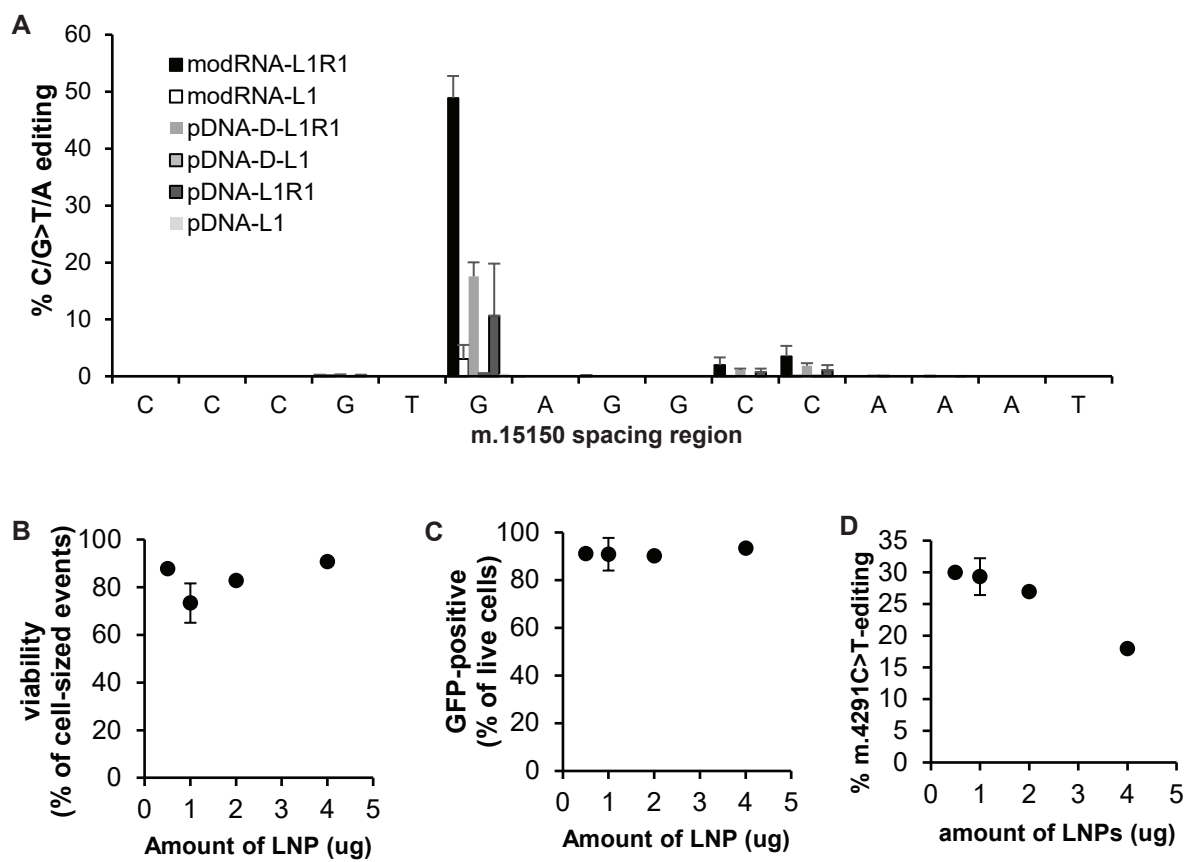

Figure S4

Supplement: S4 Fig — (A) Percent G > A or C > T editing in the m.15150 spacing region in liver organoids six days after electroporation with modRNA (N = 4), DNA plasmids (N = 3) or double DNA plasmid (N = 2) amounts for L1-only or L1R1-combination constructs. Illumina NGS. (B) Cell viability (percent of DAPI-negative cell-sized events) three days after LNP-transfection of fibroblasts with different amounts of modRNA encoding DdCBE-4291-L2R2. (C) Transfection efficiency (percent of GFP-positive live cells) of conditions in (B). (D) m.4291C > T editing efficiencies six days after LNP-transfection. N = 3 for 1 μg-condition only. Sanger sequencing. Raw data are provided in S1 Data. (PDF) [file pbio.3003207.s004.pdf]

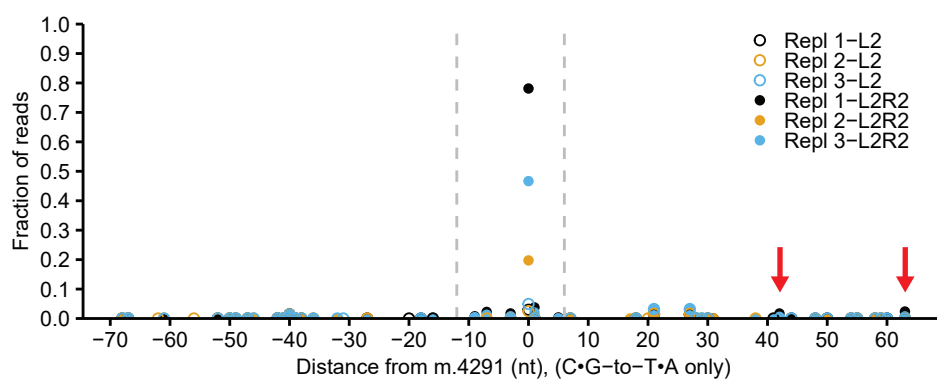

Figure S5

Supplement: S5 Fig — Two positions with likely bystander editing are indicated with red arrows. All data produced with Illumina NGS. Raw data are provided in S1 Data. (PDF) [file pbio.3003207.s005.pdf]

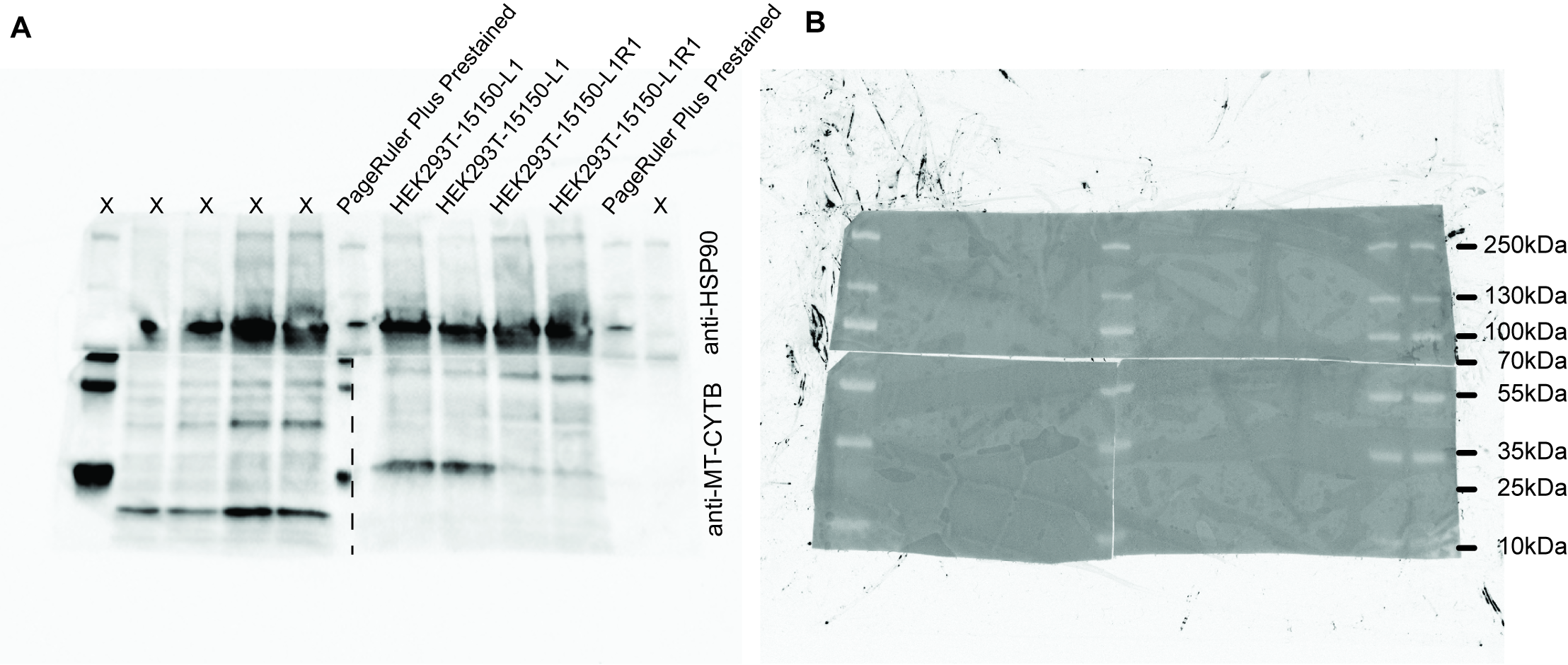

Supplement: S1 Raw Images — (A) The complete immunoblot image captured with a ChemiDoc Bio imaging system after anti-HS90 (top half) and anti-CYTB staining (bottom right portion). The bottom left portion of this blot was stained with a different antibody and not used for this study. Lanes not included in the final figures are marked with an “X” above the lane label. (B) Colorimetric image of the same blot to indicate the molecular weight marker bands. (TIF) [file pbio.3003207.s006.tif]
